# Supplementary figures and images for: Interplay between SERCA, 4E-BP, and eIF4E in the Drosophila heart
Source: PLoS One. 2022 May 19;17(5):e0267156. doi: 10.1371/journal.pone.0267156 (PMC9119464; doi:10.1371/journal.pone.0267156)

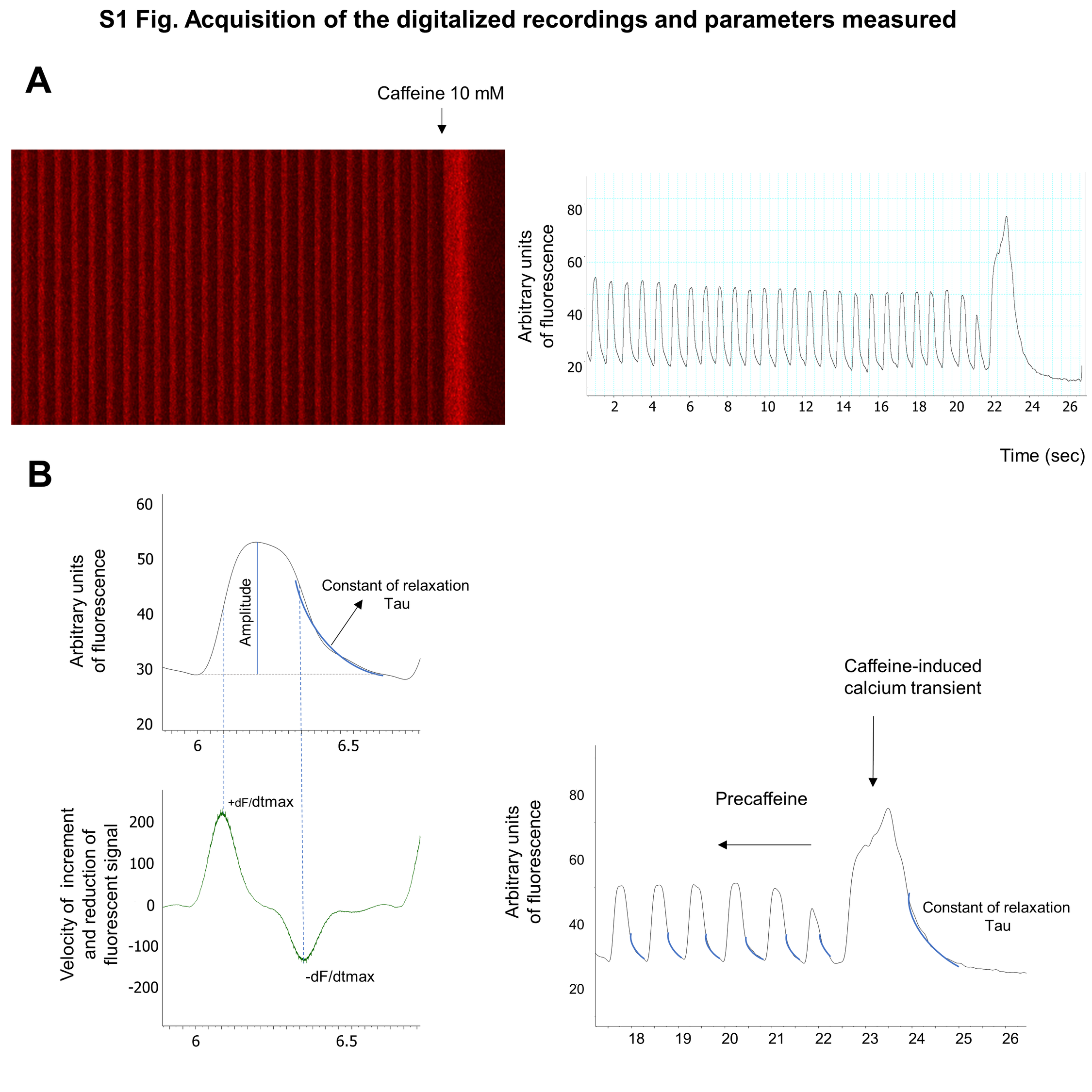

Supplement: S1 Fig — A. Image taken in the microscope (left) and digitalized recording of changes in fluorescence over time, viewed with the LabChart software (right). B Left. Example of one beat that shows all parameters measured: amplitude, expressed as relative change of fluorescence between systole and diastole, normalized by the minimum of fluoresce during diastolic period (F-F0/F0). First derivative of increment and decay of the Ca2+ transient (+ΔF/dt and -ΔF/dt). Constant of relaxation Tau. Right. Caffeine-induced calcium transient shows an increment of fluorescence and prolonged relaxation compared to the pre-caffeine Ca2+ transients. (TIF) [file pone.0267156.s001.tif]

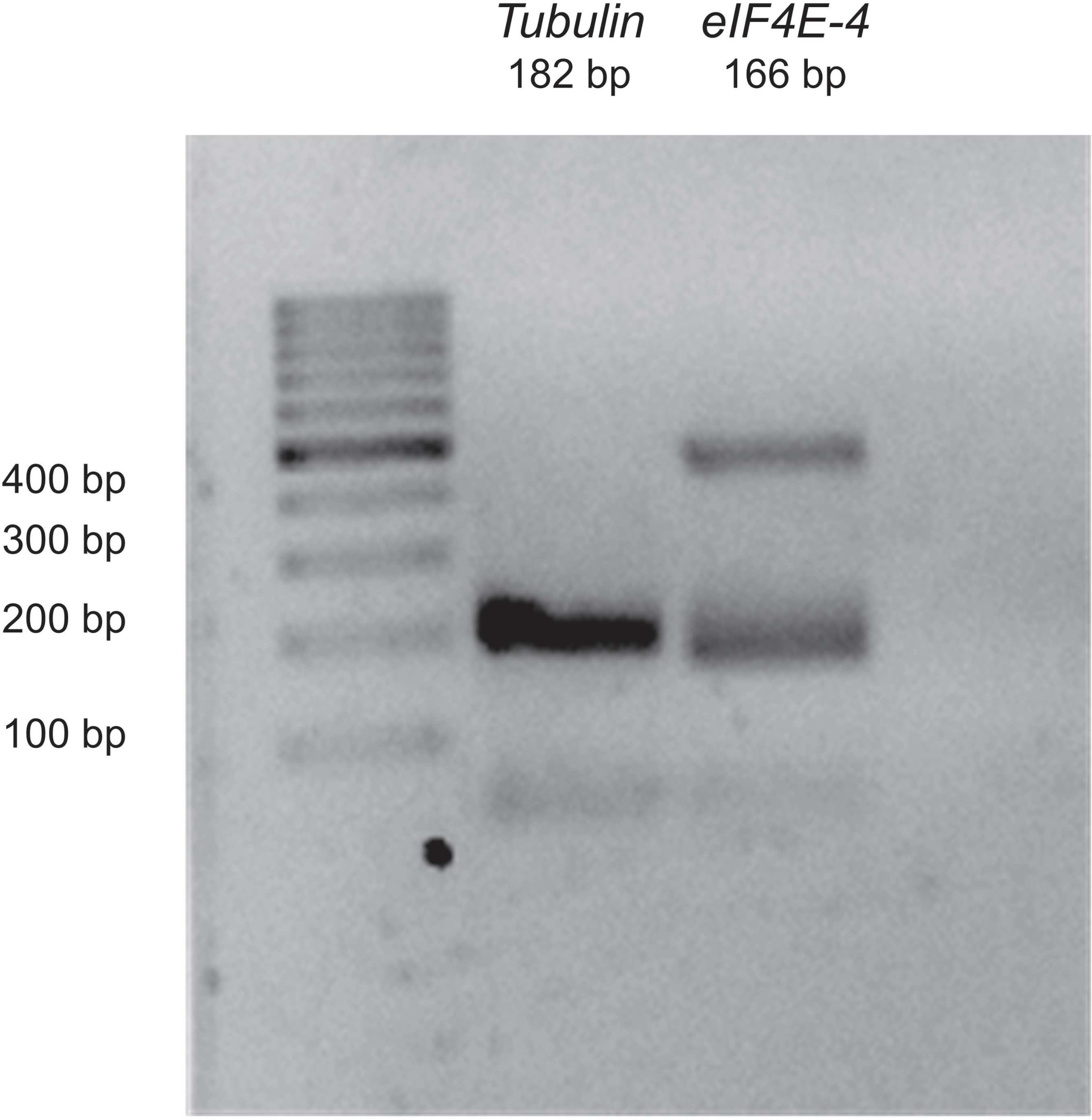

Supplement: S1 Raw image — Fragments of tubulin and eIF4E-4 were identified according to their expected size (tubulin: 182 bp; eIF4E-4: 166 bp). (TIF) [file pone.0267156.s002.tif]
