# Supplementary material for: Interplay between SERCA, 4E-BP, and eIF4E in the Drosophila heart
Source: PLoS One. 2022 May 19;17(5):e0267156. doi: 10.1371/journal.pone.0267156 (PMC9119464; doi:10.1371/journal.pone.0267156)
Supplement: S1 Table — Average results from a subset of individuals in which Tau, as well as SERCA activity were measured. Incremented SERCA activity is associated with a relaxation acceleration. All results are expressed as mean ± SEM. * p<0.05. (PDF) [file pone.0267156.s003.pdf]

S1 Table. Analysis of SERCA activity and constant of relaxation Tau.

	Tau	SERCA activity	
**control 7-days-old**	0.17	4.73	Mean
	0.04	1.07	SEM
	7.00	7.00	n
**UAS-4EBP 7-days-old**	0.08*	11.50*	Mean
	0.01	2.06	SEM
	12.00	12.00	n
